# Effects of cervical headgear appliance: a systematic review

**DOI:** 10.1590/2176-9451.20.4.076-081.oar

**Published:** 2015

**Authors:** Fernanda Pinelli Henriques, Guilherme Janson, Jose Fernando Castanha Henriques, Daniela Cubas Pupulim

**Affiliations:** 1 PhD resident, Universidade de São Paulo, School of Dentistry, Bauru, São Paulo, Brazil; 2 Full professor, Universidade de São Paulo, School of Dentistry, Bauru, São Paulo, Brazil

**Keywords:** Angle Class II malocclusion, Extraoral traction appliances, Orthodontic appliances, Removable orthodontic appliances, Orthopedic appliances

## Abstract

**OBJECTIVE:**

Although much has been investigated about the effects of cervical headgear, there remains some controversy. Therefore, the objective of this systematic review is to disclose the actual effects of the cervical headgear appliance, based on articles of relevant quality.

**METHODS:**

A literature review was conducted using PubMed, Web of Science, Embase, Scopus and Cochrane databases. Inclusion criteria consisted of human studies written in English; published between 1970 and 2014; in which only the cervical headgear was used to correct Class II malocclusion; prospective or retrospective; with a clear description of cervical headgear effects; with a sample size of at least 15 individuals. No comparative studies, clinical cases or cases with dental extractions were included and the sample should be homogeneous.

**RESULTS:**

Initially, 267 articles were found. A total of 42 articles were selected by title and had their abstracts read. Finally, 12 articles were classified as with high quality and were used in this systematic review.

**CONCLUSIONS:**

The cervical headgear appliance proved efficient to correct Class II, Division 1 malocclusion. Its effects consisted in correction of the maxillomandibular relationship by restriction of maxillary anterior displacement; distalization and extrusion of maxillary molars; and slight maxillary expansion.

## Abstract (pt)

**OBJETIVO:**

embora muitos estudos investiguem os efeitos do AEB cervical, ainda há algumas controvérsias sobre os seus efeitos. Portanto, o objetivo desta revisão sistemática é divulgar os efeitos reais do aparelho extrabucal cervical, com base em artigos com qualidade.

**MÉTODOS:**

os artigos foram pesquisados por meio das bases de dados PubMed, Web of Science, Embase, Scopus e Cochrane. Os critérios de inclusão consistiram em: estudos em humanos escritos em inglês; publicados entre 1970 e 2014; apenas o AEB cervical foi utilizado para corrigir má oclusão de Classe II; prospectivos ou retrospectivos; com uma descrição clara dos efeitos do aparelho; com um tamanho de amostra de pelo menos 15 indivíduos. Estudos comparativos, relatos de caso ou casos com extrações não foram incluídos e a amostra deveria ser homogênea.

**RESULTADOS:**

inicialmente, 267 artigos foram encontrados; e 42 artigos desses foram selecionados pelo título, tendo seus resumos lidos. Por fim, 12 artigos foram classificados como de alta qualidade e foram utilizados na presente revisão sistemática.

**CONCLUSÕES:**

o aparelho extrabucal cervical foi eficiente para corrigir a má oclusão de Classe II divisão 1. Seus efeitos são correção da relação maxilomandibular, com restrição do deslocamento anterior da maxila; distalização e extrusão dos molares superiores e ligeira expansão maxilar.

## INTRODUCTION

Growing patients can benefit from the use of the cervical headgear appliance to correct Class II, Division 1 malocclusion, although treatment effect is intimately related to patient's compliance and motivation. This protocol has been used for decades and has shown good results, providing orthopedic and orthodontic effects depending on the magnitude of force, time of daily use and patient's age.^1,2^

Although the use of cervical headgear has been currently decreasing, especially because of the development of mini-implants^3^ and the increase in the use of fixed functional appliances,^4^^-^ 7 it is still useful for specific Class II malocclusions with predominance of maxillary and/or dentoalveolar maxillary protrusion.

Studies have reported a variety of dentoskeletal effects produced by the cervical headgear, which are somewhat diverging. Therefore, this systematic review aimed to elucidate which are the actual effects of this treatment on Class II malocclusions.

## MATERIAL AND METHODS

By using the terms 'effects', 'cervical' and 'headgear', a computerized search was performed on the following electronic databases: PubMed, Scopus, Web of Science, Embase, and Cochrane (Table 1).

**Table 1.** Database research results.

Database	Results	Articles selected	Articles included
PubMed	72	19	07
Cochrane	07	00	00
Web of Science	68	02	00
Embase	36	01	00
Scopus	84	10	03
Hand searching		10	02
Subtotal		42	
Duplicate articles		30	
Total			12

Only the articles meeting the following criteria were selected for inclusion and analysis: human studies published in English between 1970 and 2014; prospective or retrospective studies, with a clear description of the effects of cervical headgear with sample size of at least 15 individuals; a homogeneous sample; studies in which only the cervical headgear appliance was used to correct Class II malocclusion. Exclusion criteria comprised comparison studies between appliances; case reports; studies on patients who used fixed appliances concurrently with cervical headgear and on patients who were treated with extractions. Duplicate articles were eliminated.

Initially, the articles were selected by titles. Subsequently, the abstracts of these articles were read to refine selection. If the abstracts did not contain enough information for the selection criteria, the article was fully read (Tables 2and 3).

**Table 2.** Details of studies included in the analysis

Author	Initial age	Daily use	n	Malocclusion
Wieslander L, Buck DL^10^(1974)	9 years	12 to 14 h	28	Class II, division 1
Wieslander L^1^(1975)	8 years	12 to 14 h	23	Class II, division 1
Kirjavainen M, Kirjavainen T, Haavikko K^13^ (1997)	9.3 years	12 to 14 h	40	Class II, division 1
Kirjavainen M, Kirjavainen T, Humrmerinta K, Haavikko K^14^ (2000)	9.3 years	12 to 14 h	40	Class II, division 1
Ashmore JL, et al^17^(2002)	Not described	14 h	36	Class II, division 1
Kirjavainen M, Kirjavainen T^12^ (2003)	9.1 years	12 to 14 h	40	Class II, division 1
Lima Filho RM, Lima AL, Oliveira Ruellas AC^8^ (2003)	10.5 years	12 to 14 h	40	Class II, division 1
Mantysaari R, Kantomaa T, Pirttiniemi P, Pykalainen A^9^ (2004)	7.6 years	8 to 10 h	68	Class II, division 1
Godt A, Kalwitzki M, Goz G^16^ (2007)	10.9 years	Not described	247	Class II, division 1
Kirjavainen M, Hurmerinta K, Kirjavainen T^11^ (2007)	9.1 years	12 to 14 h	40	Class II, division 1
Godt A, Berneburg M, Kalwitzki M, Göz G^15^ (2008)	11 years	14 h	119	Class II, division 1
Alió-Sanz J, et al^18^(2012)	8 years	12 to 14 h	79	Class II, division 1

**Table 3.** Justification for inclusion of selected articles.

Author	Article	Effects
Wieslander L, Buck DL^10^ (1974)	Physiologic recovery after cervical traction therapy	Class II malocclusion corrected by distal movement of maxillary molars. Mandibular rotation was also present and maxillary growth was redirected. Changes remained stable.
Wieslander L^1^ (1975)	Early or late cervical traction therapy of Class II malocclusion in the mixed dentition	The use of cervical headgear was more efficient in terms of skeletal changes in early mixed dentition. ANB angle decreased during the same period.
Kirjavainen M, Kirjavainen T, Haavikko K^13^ (1997)	Changes in dental arch dimensions by use of an orthopedic cervical headgear in Class II correction	Class II malocclusion corrected by improving overjet and keeping overbite unchanged. There was an increase in upper arch width and, as a result, lower arch as well. Upper arch length also increased.
Kirjavainen M, Kirjavainen T, Humrmerinta K, Haavikko K^14^ (2000)	Orthopedic cervical headgear with an expanded inner bow in Class II correction	All patients had Class II malocclusion successfully corrected. There was restriction of forward maxillary displacement and normal mandibular growth expression.
Ashmore et al^17^ (2002)	A 3-dimensional analysis of molar movement during headgear treatment	Class II malocclusion corrected by distalization with extrusion of maxillary molars and arch expansion.
Kirjavainen M, Kirjavainen T^12^ (2003)	Maxillary expansion in Class II correction with orthopedic cervical headgear. Posteroranterior cephalometric study	Malocclusion was corrected and Class I relationship reestablished in all cases. There was maxillary expansion. As a result of maxillary expansion, there was spontaneous mandibular increase.
Lima Filho RM, Lima AL, Oliveira Ruellas AC^8^ (2003)	Mandibular changes in skeletal Class II patients treated with Kloehn cervical headgear	Skeletal Class II malocclusion correction was effective and stable. The ANB angle improved, there was restriction of maxillary displacement and mandibular rotation, in addition to extrusion of maxillary molars.
Mantysaaari R, Kantomaa T, Pirttiniemi P, Pykalainen A^9^ (2004)	The effects of early headgear treatment on dental arches and craniofacial morphology: a report of 2 years randomized study.	There was an increase in maxillary and mandibular arch length and width. The use of cervical headgear proved effective to treat moderate crowding during early mixed dentition.
Godt A, Berneburg M, Kalwitzki M, Göz G^15^ (2008)	Cephalometric analysis of molar and anterior tooth movement during cervical headgear treatment in relation to growth patterns	There was extrusion of maxillary molars and mandibular rotation in patients with good growth pattern.
Kirjavainen M, Hurmerinta K, Kirjavainen T^11^ (2007)	Facial profile changes in early Class II correction with cervical headgear	Cervical headgear proved effective to correct Class II malocclusion, as it minimized overbite regardless of patient’s growth pattern.
Godt A, Kalwitzki M, Goz G^16^ (2007)	Effects of cervical headgear on overbite against the background of existing growth patterns	Class II malocclusion was corrected by the cervical headgear. There was extrusion of maxillary molars. Treatment was followed by a decrease in maxillary convexity. There was an increase in lip seal.
Alió-Sanz J et al^18^ (2012)	Effects on the maxilla and cranial base caused by cervical headgear: A longitudinal study	There was restriction of maxillary displacement in relation to the cranial base, in addition to retrusion of the A point.

The selection process was independently conducted by two researchers in the same order. Interexaminer conflicts were solved by discussion on each article so as to reach a consensus regarding which articles fulfilled the main selection criteria.

The selected articles were ultimately classified according to the following quality characteristics:^8^ number of observations, sample homogeneity, method of cervical headgear use and initial occlusal malocclusion severity.

The selected studies should present at least 15 individuals comprising the sample.^3,8^ Therefore, studies that had 15 to 20 individuals were scored as 5, those with more than 30 individuals were scored as 7, and those with more than 40 individuals were scored as 10.

Studies with a more homogeneous group were scored as 10, whereas studies lacking homogeneity were scored as 5.

Additionally, we assessed how the cervical headgear was used: studies with proper installation and adequate daily use were scored as 10, whereas failures were scored as 7 or 5.

Articles that described malocclusion severity received higher scores. However, this was not an exclusion criterion. Therefore, if the type of malocclusion was described, the article was considered acceptable (Table 4).

**Table 4.**
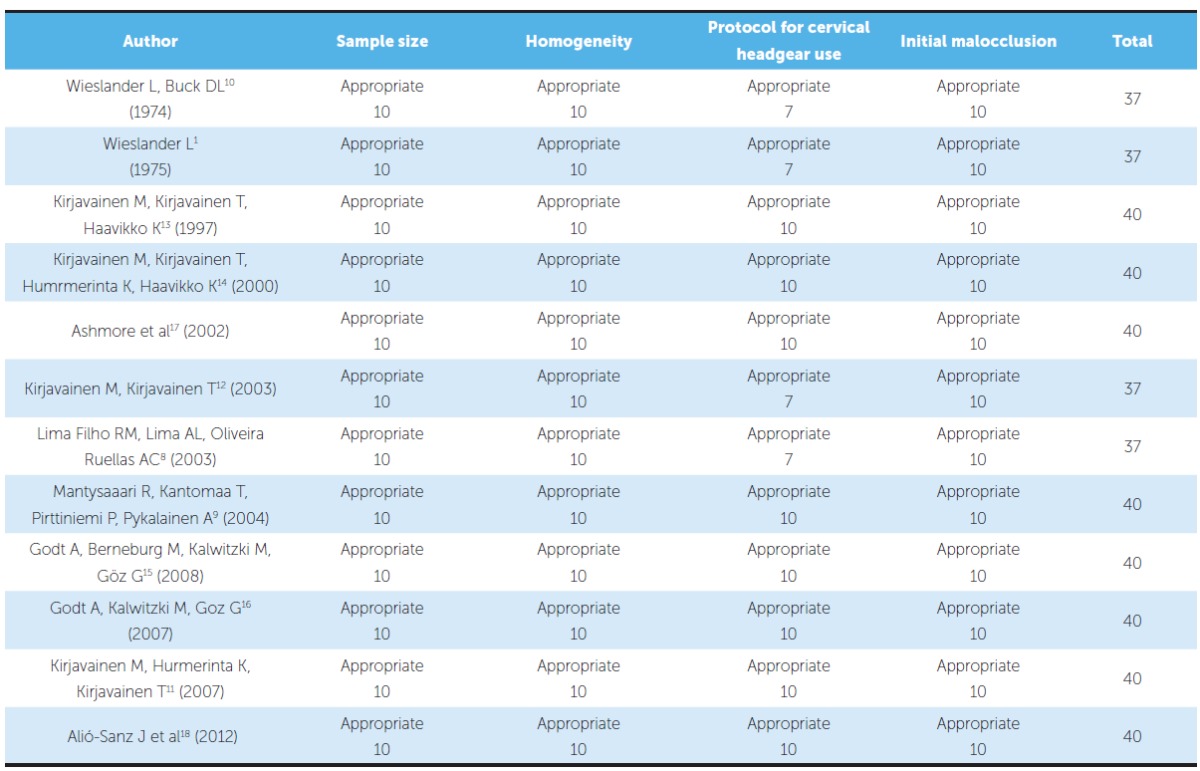
Assessment of the quality of articles selected.

The quality level of articles was assigned as follows:^8^high = total score from 30 to 40; medium = total score from 20 to 30 points; low = total score from 0 to 20.

## RESULTS

**Figure 1.**
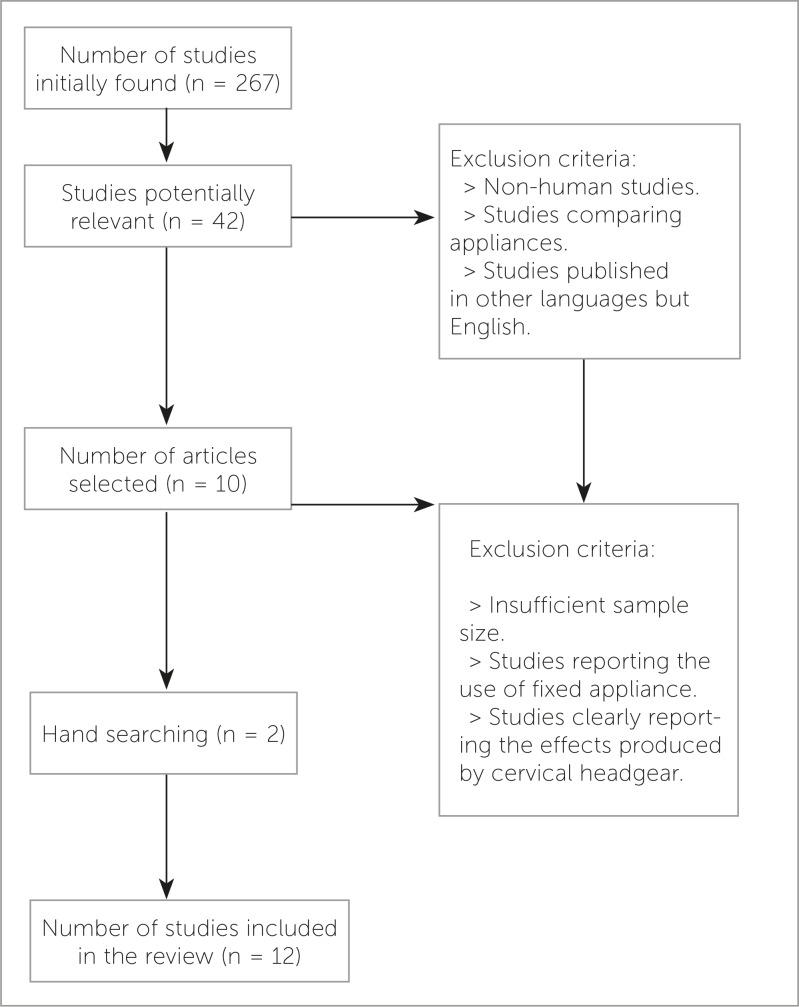
Fluxogram of database research.

After the database searching, 72 articles were found on PubMed, 7 on Cochrane, 68 on Web of Science, 36 on Embase, and 84 on Scopus (Table 1). Two articles were found by hand searching and 10 articles met the initial inclusion criteria (Fig 1). A synthesis of the information comprising the 12 selected articles is presented in Tables 2 and 3. After all analyses, 12 articles were classified with high level quality and were used in this systematic review (Table 4).

## DISCUSSION

All patients selected in the articles presented Class II, Division 1 malocclusion with a protrusive maxilla that would benefit from correction with an orthopedic cervical headgear as the only appliance.^1,8-18^

However, most articles did not describe the initial occlusal malocclusion severity and, therefore, the information in this review will be limited regarding this issue.

Class II malocclusion treatment is very difficult not only because several types of appliances can be used, but also because numerous combinations of dental and/or skeletal relationships established between the maxilla and the mandible can cause Class II malocclusion.

To avoid combined effects of several appliances, only patients treated exclusively with cervical headgear should have been considered in the selected studies.

It has also been suggested that the age at treatment onset is another critical factor.^8^ Most studies suggest starting treatment at the late mixed dentition or at the beginning of the permanent dentition to increase treatment efficiency.

The cervical headgear is supported on tubes fixed on maxillary molars bands with force ranging from 450 to 500 g on each side, and it is recommended to be used for 12 to 14 hours a day.

In the selected articles, there was extrusion of maxillary first molars, as it had been described in the 70's.^19^^,^ 20 For this reason, the use of cervical headgear alone induces bite opening and increase in vertical parameters in patients with a vertical growth pattern at the beginning of treatment.^15^ Due to molar extrusion, the cervical headgear would not be indicated for dolichofacial patients with extremely long faces, because it could worsen a profile that is already considered unpleasant.^8^^,^ 11 ^,^ 13 ^,^ 15 ^,^ 16 Notwithstanding, this would not be a reason to avoid the use of cervical headgear in patients with vertical growth.^16^

Consequently to molar extrusion, there is also mandibular clockwise rotation.^11^^,^ 15 ^,^ 16 ^,^ 17 Many researchers have found that the mandible rotates backwards and the mandibular plane angle increases with the use of cervical headgear.

Additionally, the cervical headgear promoted slight expansion of the upper arch, obtained by the expansion introduced in the inner bow of about 8 to 10 mm, which favors alignment of maxillary teeth.^13^^,^ 14 This maxillary expansion may be eventually accompanied by mandibular arch expansion^12^ and creates excellent conditions for the mandible to grow to a full extent, helping to correct Class II malocclusion.

Another headgear effect, described by the articles, was the improvement of the maxillomandibular relationship by means of maxillary repositioning.^1,9-17^ In other words, there was restriction of forward and downward maxillary displacement and normal mandibular growth expression, compensating the initial overjet that patients presented before treatment.^1,9,14^ This was especially observed in the early mixed dentition.^1,9^

All articles also showed improvements of molar relationship, that is, all patients initially found with Class II molar relationship ended up with Class I molar relationship. Therefore, there was actual distalization of maxillary molars. However, because initial anterior-posterior malocclusion severity was not specified in most articles, the amount of distalization could not be determined.^1,8-18^

All articles selected showed that patient's compliance and motivation are essential to correct Class II malocclusion.^1,8,15,16^ Nevertheless, no article reported patient exclusion due to lack of compliance, which is especially difficult with an extraoral appliance due to esthetic implications.

The orthodontist plays a great role in motivating the patients to use the appliance.^2^ If there is a good level of compliance, the favorable results demonstrated by this review can be obtained.

## CONCLUSIONS

The effects of the cervical headgear were as follows:

» Effective correction of Class II, Division 1, malocclusion.
» Correction of maxillomandibular relationship by restriction of maxillary anterior displacement.
» Distalization and extrusion of maxillary molars.
» Slight maxillary expansion.
